# Effects of Some Lamiaceae Species on NO Production and Cell Injury in Hydrogen Peroxide-induced Stress

**DOI:** 10.22037/ijpr.2019.1100685

**Published:** 2019

**Authors:** Hamed Parsa Khankandi, Sahar Behzad, Faraz Mojab, Mohammad Mahdi Ahmadian-Attari, Shamim Sahranavard

**Affiliations:** a *Department of Pharmacognosy, School of Pharmacy, Shahid Beheshti University of Medical Sciences, Tehran, Iran.*; b *Evidence-based Phytotherapy and Complementary Medicine Research Center, Alborz University of Medical Sciences, Karaj, Iran. *; c *Traditional Medicine and Materia Medica Research Center, Shahid Beheshti University of Medical Sciences, Tehran, Iran.*; d *Department of Traditional Pharmacy, School of Traditional Medicine, Shahid Beheshti University of Medical Sciences, Tehran, Iran.*

**Keywords:** Scutellaria, Nepeta, PC12 cells, Griess assay, Nitric oxide, Oxidative stress

## Abstract

Nitric oxide (NO) is a key mediator that plays an important role in pathogenesis of various chronic diseases like Alzheimer’s disease and Parkinson’s disease. Additionally, there is a great attitude for finding natural compounds, which could control and inhibit NO production in pathological conditions. Therefore, we were encouraged to investigate the effects of some Lamiaceae species on NO production and cell injury during oxidative stress in PC12 cells. In this study, cell death determined by MTT assay and NO levels were evaluated using Griess assay. PC12 cells were exposed to total metanolic extracts of three *Scutellaria* and one *Nepeta* species. The results revealed that *Nepeta laxiflora *(*N. laxiflora*) could protect PC12 cells from hydrogen proxide-induced oxidative stress and all of the plants inhibited NO production in that condition except *Scutellaria tournefortii *(*Sc. tournefortii*). In addition, *Scutellaria multicaulis *(*Sc. multicaulis*) was meanwhile subjected to fractionation using different organic solvents. The dichloromethan and ethyl acetate fractions of *Sc. multicaulis* could protect PC12 cells from oxidative stress injury. However, NO production was restrained by the hexane and dichloromethane fractions. Considering the results, *N. laxiflora*, *Scutellaria nepetifolia* (*Sc. nepetifolia*)*,* and *Sc. multicaulis* are good candidates for further investigations in neuroprotection and anti-inflammation studies.

## Introduction

Nitric oxide (NO) is a gaseous mediator produced by oxidation of L-arginine. Remarkably, three types of nitric oxide synthase (NOS) enzymes have been identified and two of them, neuronal and endothelial isoforms (nNOS, eNOS), are constitutive Ca+-dependent and act in physiological conditions. On the other hand, the inducible form (iNOS) can produce NO increasingly in pathological cellular environments (1). Classically, NO signaling was attributed to cyclic guanosine monophosphate (cGMP) especially in endothelial cells. However, recent researches reveal the effects of NO on other pathways. So it can be stimulated by various factors and in conditions, such as nitrosative stress it promotes apoptosis either dependent or independent on mitochondria (2).

Additionally, oxidative/nitrosative stress (ONS) is defined as an imbalance between pro-oxidants (reactive oxygen and nitrogen species) and antioxidants in favor of the former. Over the last decades, numerous studies have shown the key role of ONS in variety of pathologies, including chronic degenerative disease of the brain (3). Moreover, the production of nitric oxide (NO) and several pro-inflammatory factors increase in some pathological conditions and promote neuroinflammation. Notably, the neuroiflammation has a central position in neurodegenerative diseases such as Alzheimer’s disease and Parkinson’s disease (4). Furthermore, NO by changing the redox state of cell microenvironments and DNA modification or mediating angiogenesis can include cancer progression (1). Besides, in asthma pathogenesis, recent studies support the key roles of NO and reactive nitrogen species (RNS) (5). 

Considering foregoing matters, there is a huge interest in discovery of drugs or lead compounds, which could control and inhibit NO production especially in pathological conditions. Besides, some of the plant extracts are good candidates as a source for discovery of such compounds due to their antioxidant and anti-inflammatory properties. Usually these extracts have main compounds related to phenolic structures such as flavonoids (6, 7).

Notably, Lamiaceae is one of the largest flowering plant families that has 47 genera; and 371 species, sub-species, and varieties in Iran. Moreover, the plants in Lamiaceae have a wide range of biological activities and diversity in phytochemical classes such as terpenes, flavonoids, and phenolic acids (8, 9). According to a review, Lamiaceae was the most appeared plant family in Iran ethnopharmacological studies (9).

In addition, genera *Nepeta* and *Scutellaria* from Lamiaceae, have shown promising effects in antioxidant and anti-inflammation bioassays, and both of them are rich sources of phenolic and terpene compounds (10, 11).

In the present study, we investigated the inhibitory effect of total extracts and fractions of three plants from *Scutellaria* genus (two of them are endemic to Iran) and one Iran endemic plant from *Nepeta* genus, in PC12 cells, stressed by hydrogen peroxide.

## Experimental

*Chemicals*

Dulbecco’s modified Eagle’s medium (DMEM), fetal bovine serum (FBS), trypsin-EDTA solution, penicillin/streptomycin 100 units, dimethylsulfoxide (DMSO), and all other fine chemicals were obtained from Merck, Germany. 3-(4,5 dimethylthiazole-2-yl)-2,5-dimethyl tetrazolium bromide (MTT) and trypan blue were purchased from Sigma (St Louis, MO).

*Plant materials*

Three endemic plants including *Scutellaria nepetifolia* Benth., *Sc. multicaulis subsp. multicaulis* Boiss., and *Nepeta laxiflora* Benth. were collected in mid July 2016 from Hamedan province and another plant *Sc. tournefortii *Benth. was collected in May 2016 from Golestan province of Iran. All plants were identified by botanists at Tehran University of Medical Sciences (TUMC) and Shahid Beheshti University of Medical Sciences, Tehran, Iran. A voucher specimen of each species is deposited at respective herbarium.

*Preparation of extracts*

Aerial part of each plant was shade dried, powdered, and macerated with methanol in 1 to 10 proportion, respectively. Maceration lasted for 72 h and a fresh solvent replaced the extract every 24 h. For fractionation of the selected plant, maceration was carried out with the same process except that n-hexane, dichloromethane, ethyl acetate, and methanol were used as solvents, respectively. All extracts and fractions were collected and concentrated by rotary evaporator Heidolph 4000 (Schwabach, Germany) at room temperature, to remove all solvent residuals.

*Cell culture and treatment*

PC12 (rat pheochromocytoma) cells obtained from Pasteur Institute (Tehran, Iran), were grown in DMEM enriched by 10% FBS, supplemented with 100 unit/mL penicillin and 100 mg/mL streptomycin and maintained at 37 °C in a humidified atmosphere (90%) containing 5% CO_2_.

Plant extracts and fractions were dissolved in dimethyl sulfoxide (DMSO) to make stock solutions. The stock solutions and hydrogen peroxide (H_2_O_2_) were diluted with DMEM to gain interested concentrations prior to use. All of the experiments involving exposure to H2O2 and measurement of NO were performed in serum-free DMEM to avoid rapid H_2_O_2_ degradation by antioxidants and interference with Griess assay by FBS (12, 13).

**Figure 1:**
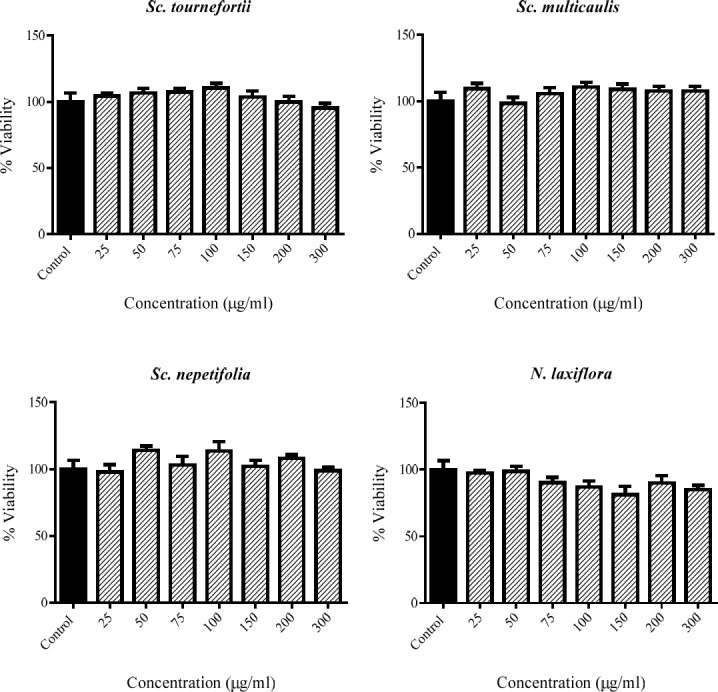
The viability percent of PC12 cells that treated with various concentration of tested plants for 24 h. Data were expressed as percentage of control group mean absorbance (Viability%) and represent as mean ± SEM (n = 6)

**Figure 2:**
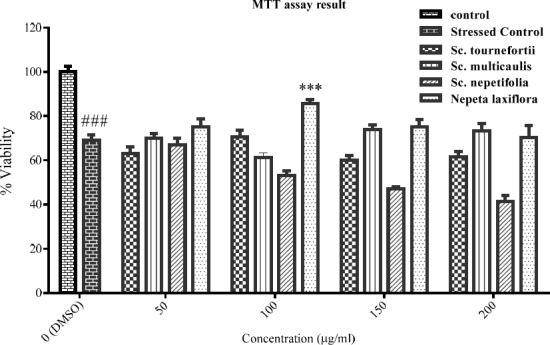
The viability percent of PC12 cells that pre-treated with various concentration of tested plants for 24 h and afterward treated with H2O2 1.5 mM for another 24 h. Data were expressed as percentage of control group mean absorbance (Viability%) and represent as mean ± SEM (n = 6). ### and ***p < 0.001 compared to control and stressed-control group, respectively

**Figure 3:**
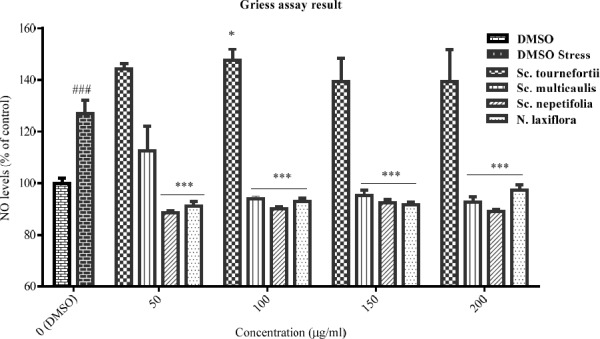
The effect of total extracts on NO production was assessed in PC12 cells that treated with H2O2 1.5 mM for 24 h. Data were expressed as percentage of control group mean absorbance (% of control) and represent as mean ± SEM (n = 6). ### and ***p < 0.001 (*p< 0.05) compared to control and stressed-control group, respectively

**Figure 4:**
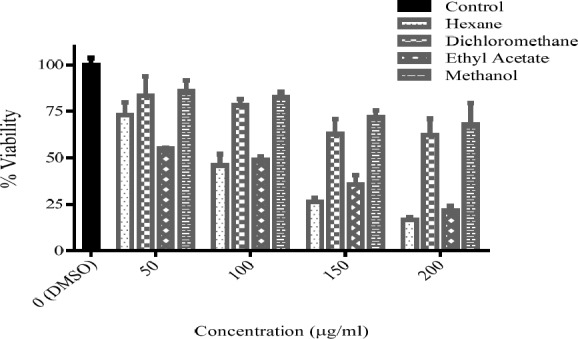
The viability percent of PC12 cells that treated with various concentration of *Sc. multicaulis *fractions for 24 h. Data were expressed as percentage of control group mean absorbance (Viability%) and represent as mean ± SEM (n = 6)

**Figure 5:**
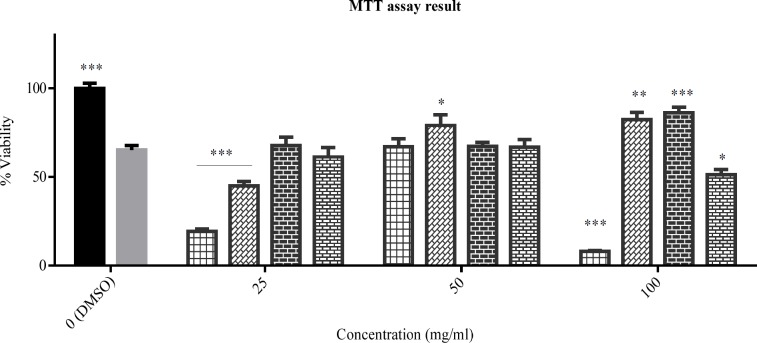
The viability percent of PC12 cells that pre-treated with *Sc. multicaulis* fractions for 24 h and afterward treated with H_2_O_2_ 1.5 mM for another 24 h. Data were expressed as percentage of control group mean absorbance (Viability%) and represent as mean ± SEM (n = 6). ^*^p < 0.05,^ **^p < 0.01 and^ ***^p < 0.001 compared to stressed-control group. Hex, the n-hexane fraction; DCM, the dichloromethane fraction; EtOAc, the ethyl acetate fraction; MeOH, the methanol fraction

**Figure 6:**
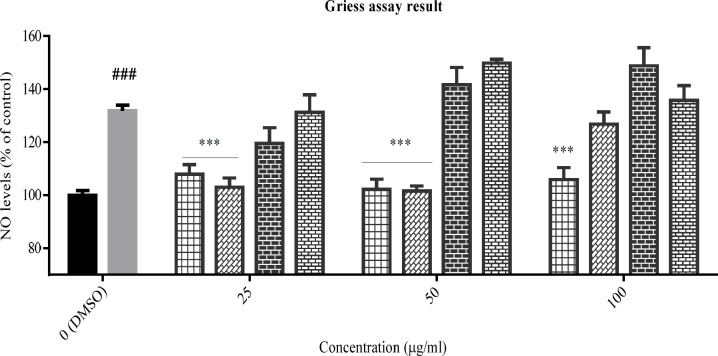
The effect of *Sc. multicaulis* fractions on NO production was assessed in PC12 cells that treated with H_2_O_2_ 1.5 mM for 24 h. Data were expressed as percentage of control group mean absorbance (% of control) and represent as mean ± SEM (n = 6). ^### ^and ^***^p < 0.001 compared to control and stressed-control group, respectively. Hex, the n-hexane fraction; DCM, the dichloromethane fraction; EtOAc, the ethyl acetate fraction; MeOH, the methanol fraction

**Table 1:** The calculated IC50 for each *Sc. multicaulis *of fractions in PC12 cells

**Fraction**	**IC** **50 ** **(μg/mL)**	**R square**
n-Hexane	105.5	0.97
Dichloromethane	n/a	n/a
Ethyl acetate	106.3	0.88
Methanol	n/a	n/a

*Cell Viability Assay*

The effect of extracts on cell viability was assessed by colorimetric method with MTT salt (14). PC12 cells were seeded in 96-well plates at a density of 35000 cells/well and incubated for 24 h. Then the medium was replaced with fresh medium containing different concentrations of each extract (25-300 μg/mL) and incubated for 24 h. Negative control wells were treated with 1% (v/v) DMSO in equal volume of medium. In the next step, the supernatant was replaced with the fresh medium, containing MTT salt (0.5 mg/mL), and incubated for 24 h. In the last step, the culture supernatant was aspirated and the formazan crystals were dissolved in DMSO. ELISA plate reader from Fisher Scientific Company (Ontario, USA) measured the absorbance of wells at 570 nm. The results presented the percentage of mean absorbance of negative control wells, considered as 100% viability. The above procedure was repeated for the fractions of the selected plant in 50 to 200 μg/mL concentration range.

*Inducing oxidative stress*

To determine the suitable concentration of H_2_O_2_, PC12 cells were seeded in 96-well plates at a density of 35000 cells/well. After 24 h incubation, the medium was freshened and all the cells were treated by 1% (v/v) DMSO and incubated for another 24 h. Then the medium was replaced by FBS-free DMEM and the cells were treated with H_2_O_2_ (1–2.6 mM) for 24 h and the cell viability was detected by the MTT method as described previously. The concentration that produced about 50% viability (EC_50_) was selected for the following tests.

*Cytoprotection by extracts and fractions as pretreatment*

For assessing extracts and fractions effect on cell viability in stressed PC12, after 24 h incubation of cells in 96-well plate (35000 cells/well), the medium was replaced and the cells were treated by DMEM-FBS10% contains extracts (25-300 μg/mL) and fractions of selected plant (50-200 μg/mL). Two groups of negative control wells were treated with 1% (v/v) DMSO in equal volume of medium. After 24 h incubation, the medium replaced by FBS free DMEM contains H_2_O_2_ (1.5 mM), except for one group of negative control (control versus stressed-control group). Twenty-four hours later, cell viability was detected by the MTT method as described previously.

*Griess assay*

A standard procedure was used to determine NO production in stressed PC12 cells (15). Briefly after seeding cells in 96-well plate and 24 h treatment with H_2_O_2_, as described above, equal volume (100 μL) of Griess reagent [1:1 mixture (v/v) of 1% sulfanilamide and 0.1% naphthylethylenediamine dihydrochloride in 5% H3PO4] and supernatant of each well was mixed and incubated for 15 min in dark place at room temperature. All extracts and fractions were employed in a process similar to that was described in the previous section. Subsequently, the absorbance of the mixture was determined at 540 nm with ELISA plate reader from Fisher Scientific Company (Ontario, USA). 

*Statistical analysis*

The data were presented as the mean ± SEM. To compare the group means, one-way analysis of variance (ANOVA), followed by Dunnett’s and Sidak’s posttests were used. All Statistical analysis were carried out by GraphPad Prism software version 6.01 from GraphPad Software Inc. (San Diego, CA, USA).

## Results

*Effect of extracts on cell viability in PC12 cells*

The cytotoxicity of the extracts in 50-100 μg/mL concentrations were assessed by MTT method in PC12 cells. There is no cytotoxicity observed for any of extracts of *Sc. nepetifolia*, *Sc. multicaulis*, *Sc.* *tournefortii, *and *N. laxiflora* in the tested range (Figure 1).

*EC*_50_ *of H*_2_ *O*_2_

The data for viability percent of cells in presence of H_2_O_2_ (1-2.6 mM) were used for fitting a nonlinear regression. According to the result, EC_50_ of H_2_O_2_ calculated 1.540 mM with 95% confidence interval of 1.507 to 1.574 mM. Therefore, the concentration of 1.5 mM was selected for inducing the oxidative stress in PC12 cells.

*Cytoprotective effect of extracts in H*_2_ *O*_2_ *-induced oxidative stress*

The PC12 cells viability was assessed in oxidative stress condition in the presence of the tested plants’ total extracts. The result (Figure 2) showed the statistically significant difference between the control and stressed-control group viability percent (*p* < 0.001), and the viability percent of stressed-control group was 69%. Hereon, the oxidative stress was effectively implied. Among the extracts, only total extract of *Nepeta laxiflora *at 100 μg/mL concentration could protect the cells against oxidative stress, significantly (*p* < 0.001).

*The extracts effects on NO production*

The inhibitory effect of the tested plants was examined by Griess assay, after pre-treatment of PC12 cells with the total extracts for 24 h and subsequently treatment with H_2_O_2_ 1.5 mM for another 24 h. According to data (Figure 3), NO production was increased in the stressed-control group compared to control cells, significantly (*p* < 0.001). The total extract of *Sc. tournefortii* showed no inhibitory effect on NO producing in any concentration and interestingly showed a significant positive effect in 100 μg/mL (*p* < 0.05). As observed in Figure 3, both *Sc. nepetifolia* and *N. laxiflora* total extracts inhibit NO production in all concentrations significantly and *Sc. multicaulis* represented similar effect in all concentrations except 50 μg/mL (*p* < 0.001).

*Effect of selected plant fractions on cell viability in PC12 cells*

Cytotoxicity of fractions of *Sc. multicaulis* was determined in 50-200 μg/mL range with a method similar to that, used for the total extracts. As expressed in Table 1, the half maximal inhibitory concentration (IC_50_) of n-hexane and ethyl acetate fractions were calculated as 105.5 and 106.3 μg/mL, respectively. Hence, methanol and dichloromethane fractions did not give under 50% viability in any concentration, IC_50 _for these fractions were estimated higher than 200 μg/mL (Figure 4). Therefore, the concentrations under 100 μg/mL were selected for oxidative stress test.

*Cytoprotective effect of Sc. multicaulis fractionss in H*_2_ *O*_2_ *-induced oxidative stress*

The PC12 cells viability was assessed in oxidative stress condition after pretreatment with tested fractions (25-100 μg/mL) for 24 h. As expected, the oxidative stress was induced successfully and the stressed-control group viability percent (65%) differed from the control group significantly (*p* < 0.001). In addition, as seen in Figure 5, the hexane fraction decreased the cell viability in 25 and 100 μg/mL (*p* < 0.01 and *p* < 0.001, respectively). The dichloromethane fraction showed protective effect in 50 and 100 μg/mL (*p* < 0.05 and *p* < 0.01, respectively) and also showed toxic effect only in 25 μg/mL.

Besides, the ethyl acetate fraction had a protective effect in 100 μg/mL significantly (*p *< 0.001) and the methanol fraction promoted cytotoxic effect in 100 μg/mL significantly (*p* < 0.05).

*The extracts effects on NO production*

After pre-treatment of PC12 cells with *Sc. multicaulis* fractions and subsequently treatment with H_2_O_2_ 1.5 mM, the inhibitory effect on NO was examined by Griess assay. Result, showed in Figure 6, proved the strong inhibitory effect of the hexane and dichloromethane fractions of *Sc. multicaulis* at 25 and 50 μg/mL (*p* < 0.001). The hexane fraction had this effect in 100 μg/mL, too (*p* < 0.001). On the other side, none of the ethyl acetate or methanol fractions could inhibit NO production in stressed PC12 cells.

## Discussion

As concerned previously, oxidative stress plays a crucial role in the pathogenesis of chronic diseases and inflammation including neurodegenerative disorders like Alzheimer’s disease (16). As a result, finding natural products with antioxidant and anti-inflammation properties is an interesting subject to recent researches. Notably, the Lamiaceae family has a great potential in drug discovery and some of its genera like *Nepeta* and *Scutellaria* are known for their remarkable antioxidant, anti-inflammatory, and neuroprotective effects (8, 11). The *Scutellaria* species have memory-enhancing, immunostimulant, anti-inflammatory, antiepileptic, and hepatoprotective properties, and were used in Chinese and Korean traditional medicine. In addition, the *Scutellaria* genus has 10 endemic species in Iran (8, 17). On the other hand, the *Nepeta* species are well-known for their antitumor, anti‐inflammatory, and antimicrobial potentials and have a great diversity in Iran. In addition 54% of Iranian species of this genera are endemic plants and Nepeta species has a great place in traditional and ethno medicines (11). 

In this study, cytoprotective and inhibition of NO effects of three endemic plants including *Scutellaria nepetifolia*, *Sc. multicaulis*, and *Nepeta laxiflora*, and another non-endemic plant named *Sc. tournefortii* have been investigated. None of the species could protect PC12 cells from oxidative stress, induced by hydrogen peroxide. However, *Sc. multicaulis* and *Sc. nepetifolia* effectively reduced NO levels in the stressed PC12 cells. In contrast, *Sc. tournefortii* increased the level of NO in oxidative stress condition. In previous studies, *Sc. multicaulis* was assessed for antioxidative, cytotoxic, and antiplasmodial effects. According to their results, it did not produce noticeable cytotoxic and antiplasmodial effects. Nevertheless, it had an average antioxidant action (18-20). There is no phytochemical study of *Sc. multicaulis* except for one study about essential oil composition that resulted in trans-Caryophyllene and Caryophyllene oxide as the main compounds (21). *Sc. tournefortii* was assessed for antioxidant effect and gave good results (22). 

Unlike Iran’s endemic *Scutellaria* spp., other species, especially *Scutellaria baicalensis *(*Sc. baicalensis*), were studied thoroughly for their neuroprotective and antioxidant effects. *Sc. baicalensis* could protect PC12 cells from oxidative stress, induced by H_2_O_2_ (23). The flavonoids from *Sc. baicalensis* protected PC12 cells from amyloid β protein-induced neurotoxicity, too (24). As these studies on *Sc. baicalensis*, and baicalein as its main component, started about two decades ago, a vast number of *in-vitro* and *in-vivo* studies on its neurodegeneration and inflammation was established completely, and are available in literature in that field (25-29). We had expected cytoprotection from *Sc. nepetifolia* and *Sc. multicaulis*; however, they only could inhibit NO production effectively and did not protect PC12 cells from oxidative stress. Nevertheless, we must notice that the extracts were applied as pretreatment in this study and additional studies to examine cytoprotection in both treatment and pretreatment mode is recommended. 

In contrast, *N. laxiflora* inhibit both cell death and NO production in stressed PC12 cells. Only one study was found in literature about *N. laxiflora* that examined antioxidant activity of essential oil which concluded that it produced a moderate effect (30). Still, there are many studies, which examine other *Nepeta *species’ effects in various pathological conditions. Süntar *et al.* (11) thoroughly reviewed biological activities of *Nepeta* spp. and highlighted that *N. flavida* and *N. sintenisii* exerted moderate antioxidant activity. Other species like *N. cataria* and *N. clarkei* produced anti-inflammatory effects. Distinctively, based on this review, there is no specific study about *Nepeta* species cytoprotective and NO reducing effect in oxidative stressed cells. By noting, in the present study, that *N. laxiflora* is an Iranian endemic plant and protects PC12 cells from stress, this species is a good subject to supplementary neuroprotection studies.
